# Development of islet organoids from human induced pluripotent stem cells in a cross-linked collagen scaffold

**DOI:** 10.1186/s13619-021-00099-z

**Published:** 2021-12-01

**Authors:** Shruti Sandilya, Shashi Singh

**Affiliations:** CSIR- Centre for Cellular and Molecular Biology, Uppal Road, Hyderabad, 500007 India

**Keywords:** Endocrine, Exocrine, Marker genes, Crosslinked collagen, Functional, Insulin release

## Abstract

Islets organoids would have value in the cell replacement therapy for diabetes apart from usual personalized drug screening routes. Generation of a large number of Islets like clusters, with ability to respond to glucose stimulation appears to be an ideal choice. In this study we have generated islet organoids with the ability to respond to glucose stimulation by insulin release. The source of the cells was an iPSC cell line differentiated into the pancreatic progenitors. These cells were assembled in matrigel or cross-linked collagen scaffold and compared for their efficacy to release insulin upon stimulation with glucose. The assembled organoids were examined by immunohistochemistry and expression of the relevant marker genes. The organoids showed expression of islet like markers in both - matrigel and crosslinked collagen scaffold. The islet organoids in both the cases showed release of insulin upon stimulation with glucose. The crosslinked collagen scaffold is quite stable and supports islet cells growth and function.

## Background

The standard available option for the management of diabetes is exogenous administration of Insulin and that is grossly inadequate to contain the disease. Insulin needs to be daily injected and its unregulated control could cause glycemic fluctuations and other side effects. The other options like pancreas transplantation and /or islet transplantation also suffer from the lack of donors and the immune-compatibility problems; lack of transplantable material remains the core issue. Derivation of the disease relevant cells from pluripotent sources is a crucial step towards the stem cell therapies for functional recuperation. Strategies to develop unlimited supply of the beta cells rely upon the differentiation potential of pluripotent cells.

Pancreas originates from the embryonic endoderm marked by the emergence of two distinctive tissue types having distinct markers, functions and morphology. The endocrine region comprises of the cells secreting hormones that maintain the euglycemic state in the organism; and an exocrine region with the acinar glands secreting digestive enzymes (Jennings et al. 2013, 2015). The rudimentary pancreas, derived from two buds from the foregut region, undergo extensive proliferation and a series of lineage differentiation to form multipotent progenitors that give rise to the ultimate complex pancreas. Each stage can be defined by essential transcription factors (Gu et al. 2004).

It has been a difficult process to obtain sustainable cultures of the pancreatic origin in humans, with intact functional characteristics. The multipotent pancreatic progenitors have also been difficult to identify in situ (Blauer et al. 2011; Houbracken et al. 2011; Grapin-Botton 2016). The multi lineage and multicellular pancreatic spheres were first generated from the ductal cells (Smukler et al. 2011).

Organoids, a three dimensional organization of cells, representing a functional organ in a miniature form in a dish; have an indefinite passaging capability along with genetic stability (Sato et al. 2009). If we could induce beta cells with an islet like organization in an organoid with its full functional capabilities, the system can be exploited not only for drug screening efficacy but also for regenerative medicine. The organoid could be a possible source of the donor tissue for the islets transplantation. With organoid development from intestine and other gut associated organs (Sato et al. 2009; Spence et al. 2011; Huch et al. 2013; McCracken et al. 2014); efforts were again made with adult pancreas and organoid clusters from pluripotent cells (Sugiyama et al. 2013, Greggio et al. 2013, Takeuchi et al. 2014, Boj et al. 2015, Hindley et al. 2016). The key was to successfully generate pancreatic progenitors which are multipotent in nature and express SOX9, PDX1 and give rise to almost all the lineages in both the exocrine and the endocrine pancreas.

Generation of beta cells basically requires close mimicking of the developmental programs of the organ that involve four stages in a culture dish: endoderm formation, posterior foregut formation, pancreatic progenitor cells and endocrine maturation (Navarro-Tableros et al. 2019; Soltania et al. 2019). Each stage is characterized by specific set of markers (Gradwohl et al. 2000; Nelson et al. 2007; Kelly et al. 2011; Rezania et al. 2013; Bonfanti et al. 2015; Nostro et al. 2015). There are a number of studies dealing with the generation of pancreatic progenitor cells with different efficiencies and also depending on the type of cells (Anderson et al. 2020).

Extracellular matrix (ECM) plays a crucial role in providing mechanical, interactional and functional support to the cells. ECM plays a role in cell proliferation, differentiation, and survival of the cells (Daley et al. 2008). Most of the organoid studies have used matrigel to support the process of organoid formation (Yin et al. 2016). Collagen has been used to entrap the pancreatic progenitors to make the organoids but these organoids lack stability, we have crosslinked collagen with oxidized gum Arabica (Sarika et al. 2014, Ragothaman et al. 2014, Rohit et al. 2021) and created stable scaffolds that appear to host the islet like clusters of the pancreas.

The present study describes successful generation of the islet spheroids from iPSC cells that express most of the markers associated with endocrine pancreas and are gluco-responsive in culture conditions. The islet like structures were assembled in presence of matrigel, collagen and GACO scaffold and a comparison was made for their sustainability and function.

## Methods

### Generation of pancreatic progenitors

iPSC cell line M1 (CCMBi001-A) generated in the lab (Jamwal et al. 2020) were maintained in DMEM:F12 (supplemented with 2 mM glutamax, 0.1 mM nonessential amino acids, 0.1 mM beta mercaptoethanol and 4 ng/ml basic fibroblast growth factor, 1% penicillin-streptomycin) over the matrigel coated plates. iPSC cells were passaged every 6–8 days using 5 nM EDTA for maintenance.

iPSC cells were aggregated to form embryoid bodies that were cultured on the matrigel coated plates. Differentiation was initiated by incubation in the basal medium (RPMI containing 0.5% knockout serum, 10 ng/m. glucose, 1% B27, 2 mM Glutamax and 1% penicillin streptomycin) with 3 μM CHIR99021 and activin A 50 ng/ml for 24 h followed by incubation in activin A 50 ng/ml for 2 days.

At this stage the cells were shifted to basal medium containing FGF7 (50 ng/ml) and ascorbic acid 250 μM for 2 days. The pancreatic progenitor differentiation was initiated by exposing the cells to basal medium containing noggin 100 ng/ml, FGF7 (25 ng/ml), 1 μM retinoic acid and phenyl dibutryl (200 nM), 1 μM dorsomorphin and ascorbic acid 250 μM for 3 days followed by reducing retinoic acid to 0.1 μM for further 2 days. Cells were stained and characterized at end of each stage for progression of the differentiation process and PP cell formation by immunostaining.

### GACO scaffolds

Collagen (0.25–1%) was crosslinked with gum Arabica oxidized to 5% (gum Arabica aldehyde) (Rohit et al. 2021). Collagen solution was mixed with gum Arabica solution in 1:1 ratio and vortexed to mix resulting in the gel formation. The assembled cells were added to the mix and dispensed in culture dish as drops. The gel was allowed to solidify and incubated in the Pancreatic Organoid Medium (POM). Plain collagen gels were made with borax buffer and similarly allowed to form drops with cells contained within.

### Spheroid formation

Early progenitors of pancreas were assembled in suspension and entrapped in the growth factor reduced matrigel, collagen or GACO gel and allowed to solidify. These aggregates were incubated in the Pancreatic Organoid Medium (POM) containing DMEM:F12 with 1% penicillin-streptomycin, HEPES 10 mM, glutamax1%,N2–1%, B-27 1%, EGF-50 ng/ml, noggin 10 ng/ml, wnt3a 25 ng/ml, A83–01500 nM, gastrin 10 nM, 1 mM acetylcysteine, R-spondin 10 ng/ml, nicotinamide 1 mM. Medium was changed every 2 days.

After day 30, few organoids were split into smaller aggregates by enzyme digestion by trypsin for 10 min or mechanical splitting (by pipetting in small amount of liquid vigorously) and cultured in the pancreatic organoid medium till they grew into larger organoids.

### Insulin release assay

Organoids were collected on day 20 and 30 were stimulated with glucose for release of insulin following protocol of Oscar and Peter (Oscar and Peter 2019). Organoids were collected in sets of 3 or 4 along with a control group for each set and incubated in glucose free POM. After 24 h of incubation with the glucose free medium, the supernatant was collected. Each set of the organoids were then fed with glucose free POM supplemented with 0 mM, 2.8 mM, 11 mM and 22 mM of glucose. The supernatant was collected post 2 h and after 24 h. The organoids in each well were then subjected to protein estimation. Culture supernatant was used for estimation of Insulin using commercial kit following the manufacturer’s instruction. (Human Insulin ELISA kit (cat # ELH-Insulin 1, RayBiotech GA). The insulin was expressed as μUnits /mg protein and normalized using control as reference.

### RNA isolation and qPCR

Total RNA was extracted using Trizol (Cat# RNAisoplus 9109 Takara Bio) and 1 μg of total RNA was reverse transcribed by a cDNA Synthesis Kit (Cat# 6110A Takara Bio) with random hexamer primer and OligodT primer. qPCR was performed for stage specific markers and islet specific markers (Table 1). The primers were custom synthesized by Bioserve India Pvt. Ltd. and optimized using a crosswise combination matrix. A total of 25 ng DNA was used for REAL time PCR with SyBR Green (Cat#4367659; Invitrogen) as indicator using Applied Biosystem (7900) HT fast real time PCR system. Glucose 6 phosphatase dehydrogenase, actin and β-2 microglobin served as internal controls. Fold changes in the gene expression were calculated by 2∆CT method. Analyses were done for the fold increase in the expression of markers in comparison with the control iPSC cells. Statistical analysis was carried out using the GraphPad prism.

**Table 1:** Sequence of Primers used for Real Time PCR

Gene	Primer F	Primer R
SOX2	5′-TGATGGAGACGGAGCTGAA	5′- GGGCTGTTTTTCTGGTTGC
*PDX1*	5′-CCTTTCCCATGGATGAAGTC	5′-TTCAACATGACAGCCAGCTC
*PAX4*	5′-ATGAACCAGCTTGGGGCTCT	5′-CTCCTTCCCACTCCCTGCCTC
*NGN3*	5′- TGTGGGTGCTAAGGGTAAGG	5′- GGCCTAAAATGAGCGCACTT
*PPY*	5′-ACCTGCGTGGCTCTGTTACT	5′-TACCTAGGCCTGGTCAGCAT
*INS*	5′-CCTTCTGCCATGGCCCTG	5′- GGCTGCGTCTAGTTGCAGTA
*NeuroD1*	5′-GAGACGCATGAAGGCTAACG	5′-CTGAACGAAGGAGACCAGGT
*mafA*	5′-CTTCAGCAAGGAGGAGGTCA	5′-TTGTACAGGTCCCGCTCTTT
*PAX6*	5′-CACTGGGGAAGGAATGGACT	5′-TTCGTGGCAAAGCTTGTTGA
*SOX9*	5-ATGAAGATGACCGACGAGCA	5′-AACTTGTCCTCCTCGCTCTC
*NKX6.1*	5′-CTTCTGGCCCGGAGTGATG	5′-GGGTCTGGTGTGTTTTCTCTTC
*B2M*	5′-GGCTATCCAGCGTACTCCAA	5′-GATGAAACCCAGACACATAGCA
*TBP*	5′-TGCACAGGAGCCAAGAGTGAA	5′-CACATCACAGCTCCCCACCA

### Immunostaining

Cells were fixed with formaldehyde and embedded in OCT solution to be frozen. The organoids were sectioned in a LEICA cryotome. The tissue sections were blocked with DPBS containing 3% BSA for 20 min at room temperature. The sections were incubated in primary antibody (Table 2) for various markers overnight at 4 °C. After washing with DPBS, the cells were incubated in appropriate secondary antibody for 45 min at room temperature, counter stained with DAPI and examined in Axioimager using appropriate filters.

**Table 2:** List of antibodies

Primary antibody	details	Secondary antibody	details
Mouse PAX6	ab78545	Donkey anti rabbit IgG H&L DYLIGHT 594 or 488	ab96921
Rabbit anti SOX17	ab224637	Donkey anti rabbit IgG H&L DYLIGHT 594 or 488	ab96921
Rabbit anti PDX 1	ab 47,267	Donkey anti rabbit IgG H&L DYLIGHT 594 or 488	ab96921
Rabbit anti somatostatin antibody	ab 183,855	Donkey anti rabbit IgG H&L DYLIGHT 594 or 488	ab96921
Goat anti Glucagon	ab36232	Donkey Anti-Goat IgG H&L (DyLight 594)	ab96933
Rabbit anti pancreatic polypeptide antibody	ab11369	Donkey pAb to Rabbit ig G Day light 488	ab96891
Rabbit anti NGN3	ab38548	Donkey pAb to Rabbit ig G Day light 488	ab96891
Mouse anti Carboxypeptidase	ab84999	Dnk pAb to anti mouse 550	ab96876
Chicken X insulin	ab3455	Donkey Anti –chickenIg Y H&L (FITC)	ab63507
Rabbit PAX4	PA1–108	Donkey pAb to Rabbit ig G Day light 488	ab96891

### Statistical analysis

All the quantitative data were analyzed using GraphPad Prism software 8.4.2/3, using paired T test or ANOVA as suitable to data. *P* values of less than 0.05 were considered significant.

## Results

### Generation of pancreatic progenitor (PP) cells

Pancreatic progenitor cells are defined by the expression of transcription factors like PDX1, NKX6.1, SOX9, etc. These cells transplanted into mice are capable of giving rise to all the derivative lineages of pancreas. So the strategy to expand a population of islet like cells would require acquiring the pancreatic progenitor cells starting from iPSC (Rezania et al. 2014).

**Fig. 1:**
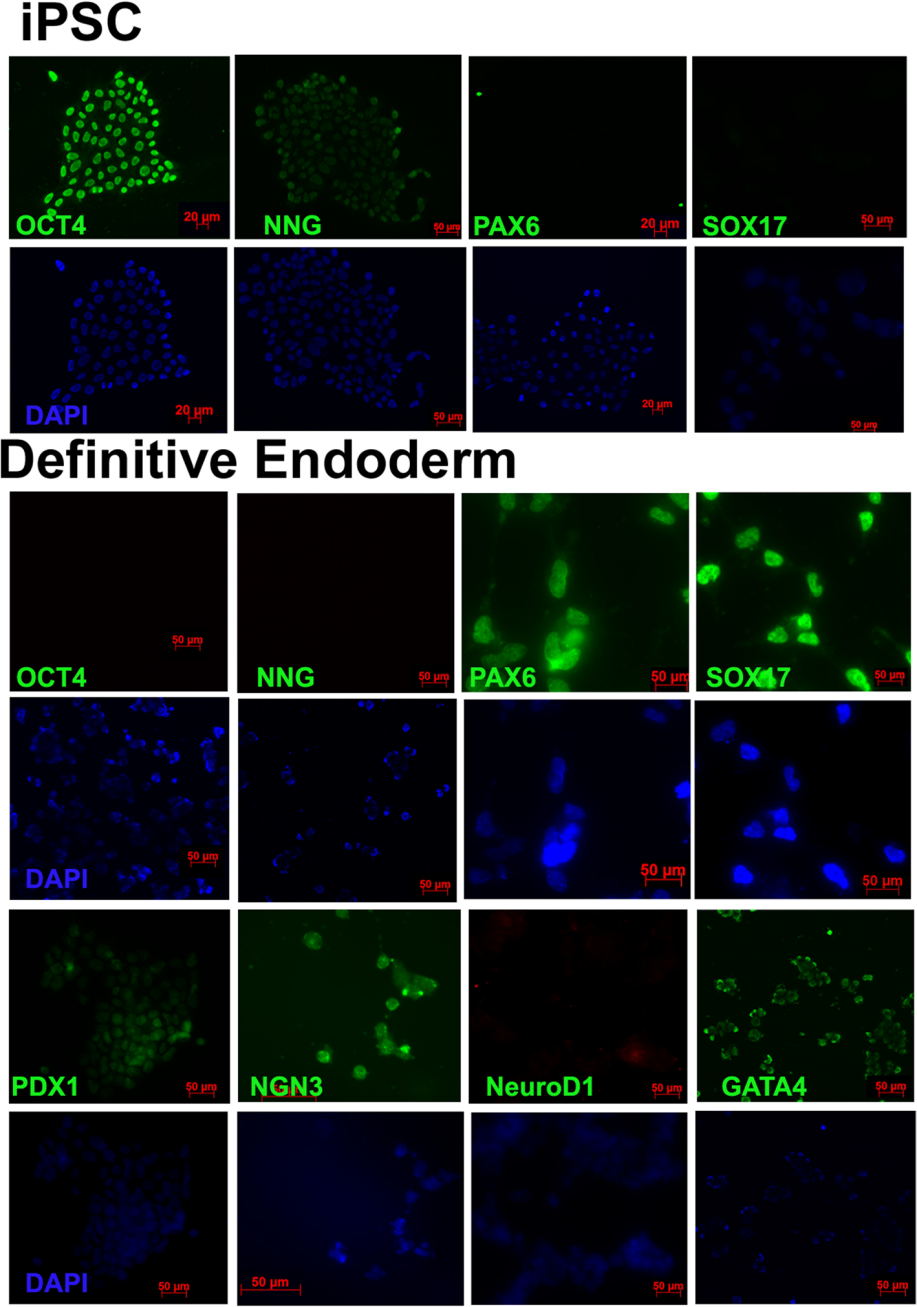
Stage-wise Immunohistochemistry of the cells undergoing differentiation. HiPSC stained for OCT4, NANOG, PAX6 and SOX17 showed expression of pluripotency markers only. Definitive endoderm show staining for PAX6, SOX17 and GATA4,and other oancretaic markers, Pluripotency markers remain negative

**Fig. 2:**
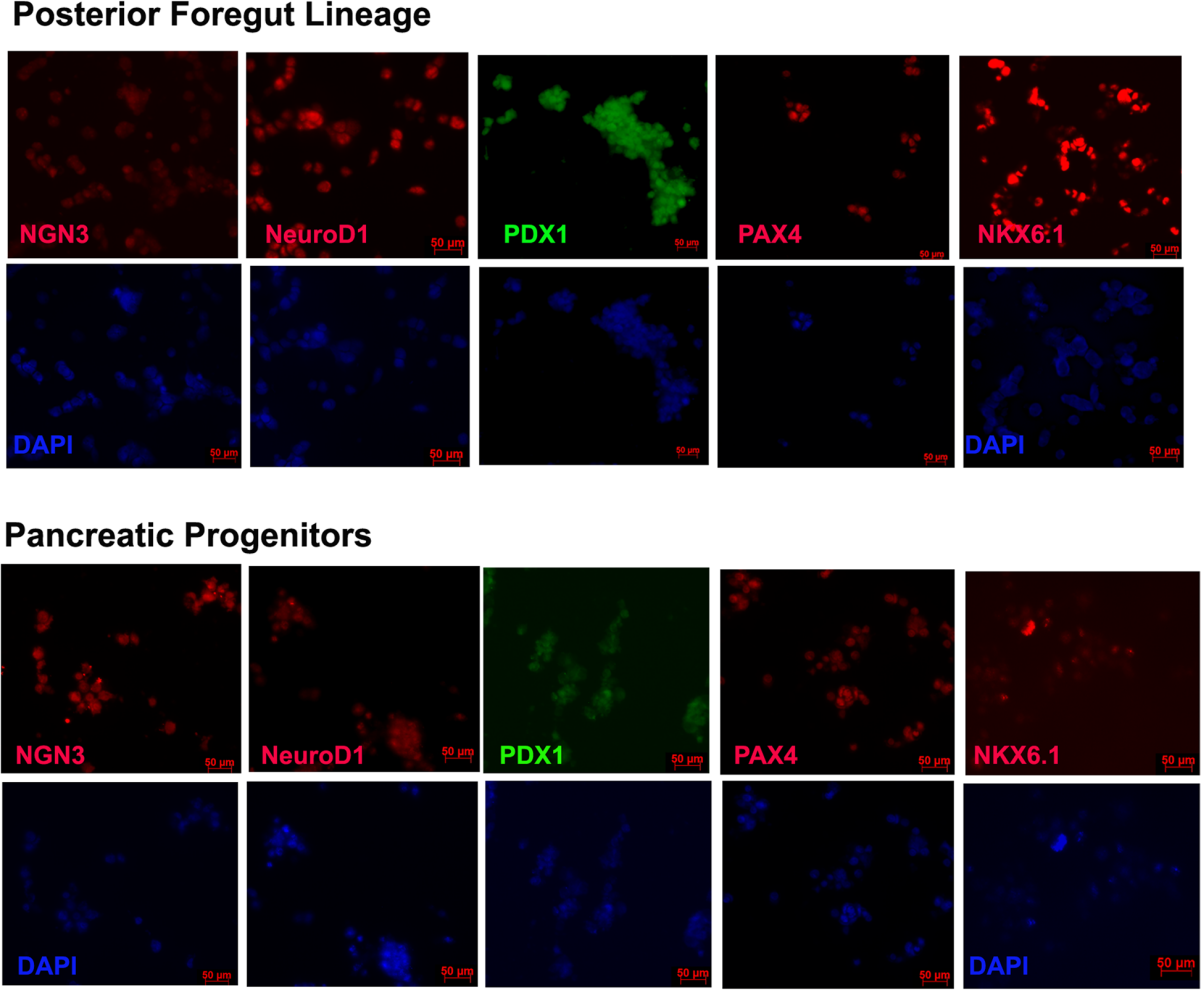
Stage wise Immunohistochemistry of the hiPSC cells undergoing differentiation. Foregut lineage cells show positive staining for PDX1 and faint staining for few progenitor markers. Pancreatic progenitor cells show positive staining for all the stage specific markers

Pancreatic organoids have been generated from the pancreatic progenitors derived from iPSCs through a series of steps. We have employed the previously described procedure with some modifications to achieve our goal (Anderson et al. 2020). iPSC cells were induced into definitive endoderm cells positive for markers GATA4, SOX17 and PAX6 using the classical pathway using Wnt activator CHIR99021 and nodal related protein activin A (Fig. 1), The cells at this stage clearly had low expression of pluripotent markers OCT4 and NANOG and expressed the definitive endoderm markers. The endoderm cells were pushed into the posterior foregut lineage in the second stage by adding epidermal growth factor to the medium, cells stained at this stage showed the expression of PDX1, and some slight positive staining for PAX4 and neuroD1. Differentiation of the foregut lineage into pancreatic progenitor cells was carried out with the addition of retinoic acid, epidermal growth factor, BMP inhibitors noggin, dorsomorphin and dibutyrl cAMP. The differentiation was confirmed by immuno-fluorescence for expression of NKX6.1, PDX1, NGN3, PAX4 and neuroD1 (Fig. 2).

**Fig. 3:**
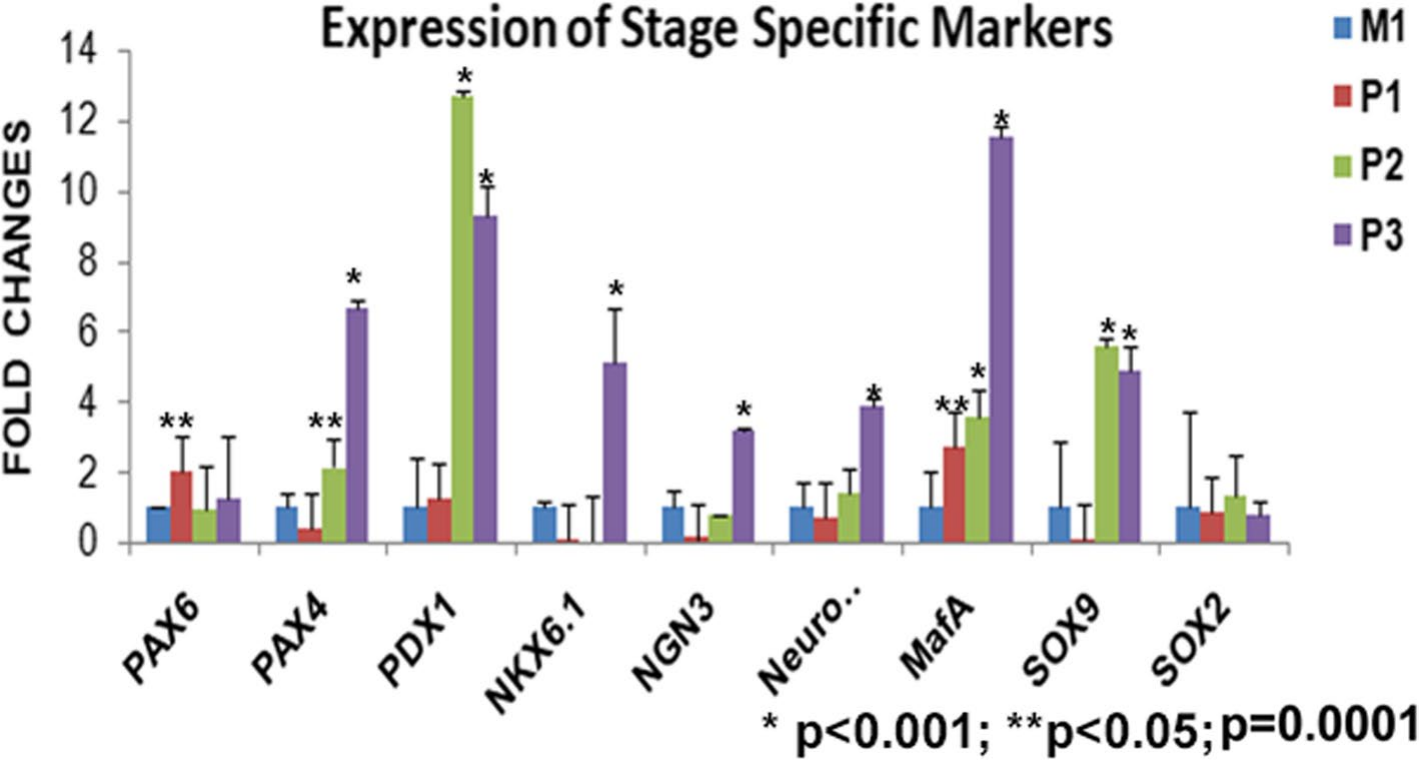
qPCR analysis of differentiation stage specific genes in cells in comparison to hiPSC M1. Stage wise expression of genes can be seen across the panel. *PAX6* is expressed in stage P1 itself and other genes start their expression at second stage P2. The levels of pancreatic progenitor markers are highest in stage P3

Staging of the cells through the process of differentiation was also confirmed by quantitative expression analysis of the genes (Fig. 3). RNA isolated from each stage was compared with the parent cell line M1 and we found that *PAX6* starts expressing at stage 1 itself with the down regulation of the pluripotent genes, other markers specific to foregut lineage or progenitors do not express significantly at this stage (*p* = 0.08). At stage 2, cells express *PDX1, MafA, PAX4* and *SOX9* manifold higher. Stage 3 cells showed significant expression of all the pancreatic progenitor markers *PDX1, PAX4, NKX6.1 NGN3, MafA*, and *Sox9 (p < 0.001)*. *SOX2* expression remains low in cells at all the stages (Fig. 3). There is temporal pattern of expression of markers as defined by the stages.

### Generation and characterization of islet spheroids

**Fig. 4:**
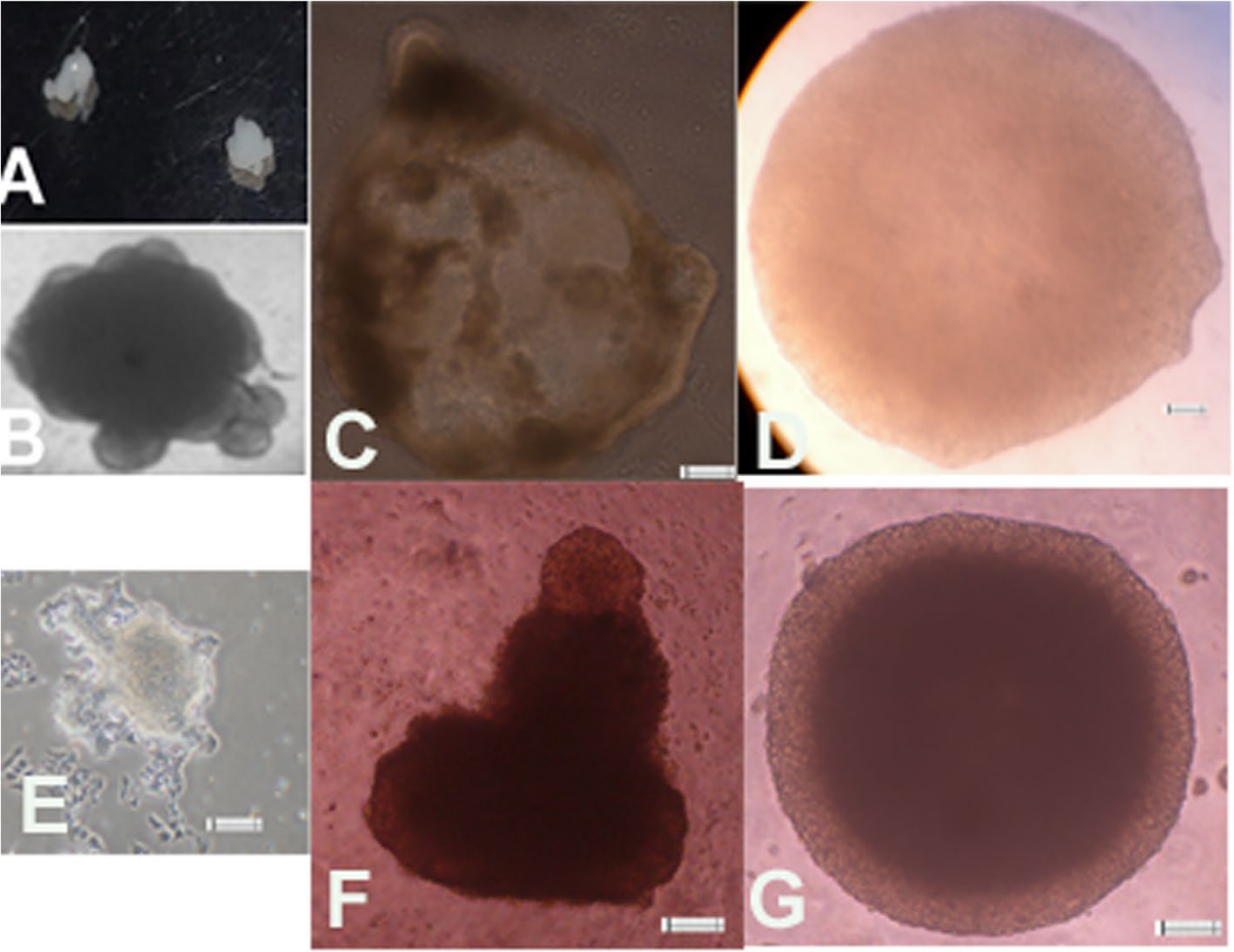
Formation of organoids from PP Cells. **A-B** Gross morphology of the organoids. **C**- Organoids as seen in microscope show a defined boundary. **D** - Organoid in a GACO scaffold. **E-G**- Organoids can be regrown from few cells after digestion or by mechanical dissociation

**Fig. 5:**
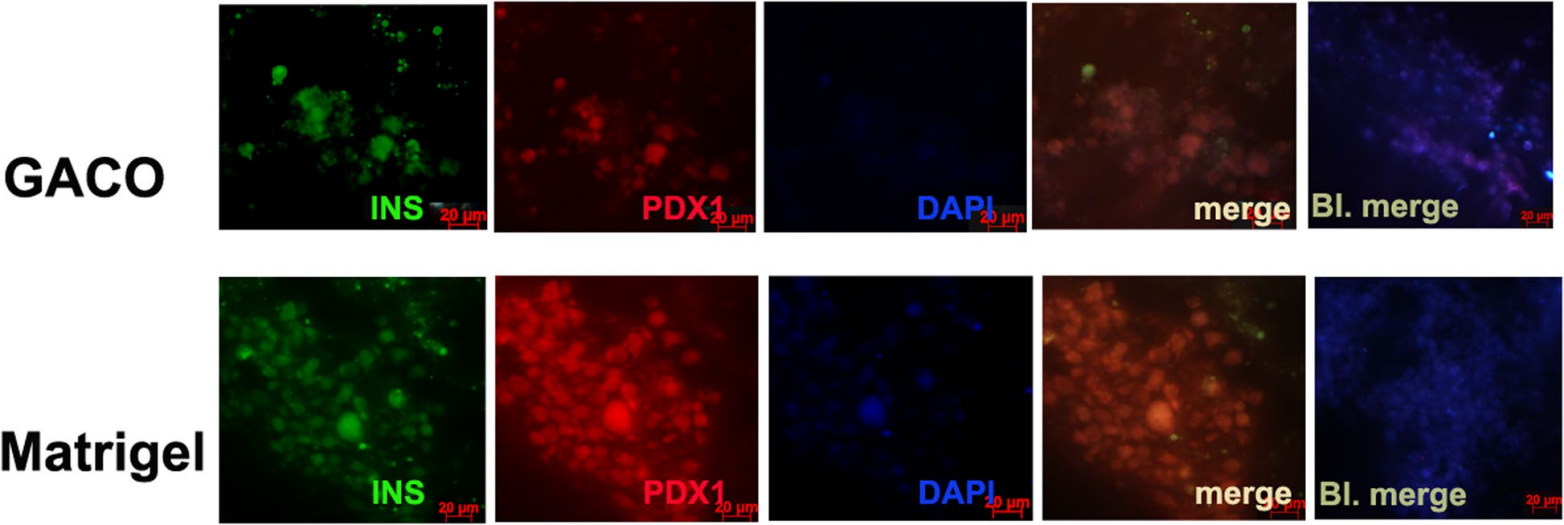
Immunohistochemistry of the organoids stained with Insulin and PDX1. Majority of the cells in cluster appear positive for PDX1 and Insulin in GACO scaffold and Matrigel

**Fig. 6:**
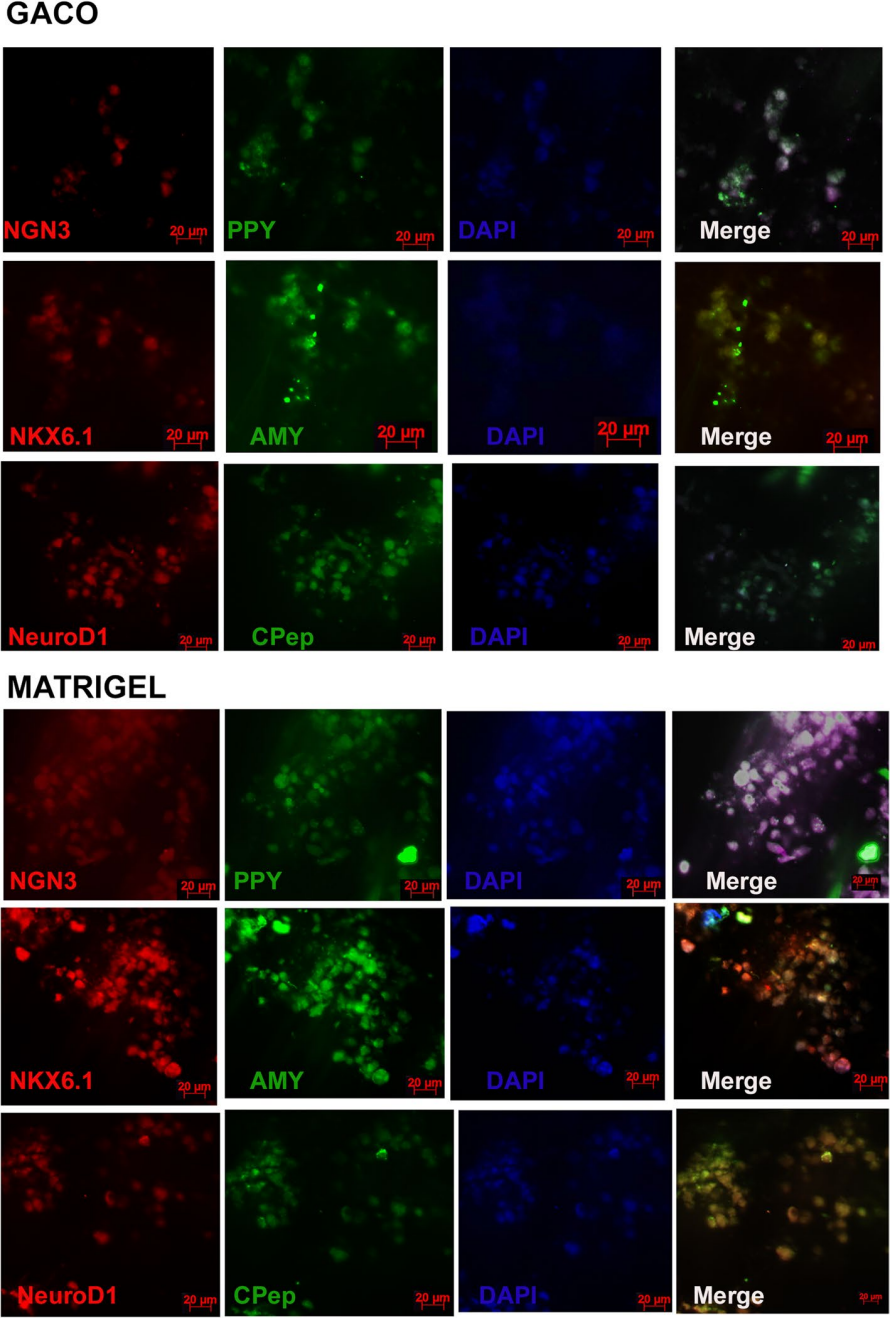
Panel showing the staining of organoid sections from the GACO gel and matrigel. Cells showed positive staining for NGN3 and PPY. Few cells appear positive for amylin among the NKX6.1 positive cells. C peptide and NeuroD1 positive cells can also be seen in organoids generated in matrigel and GACO scaffolds

**Fig. 7:**
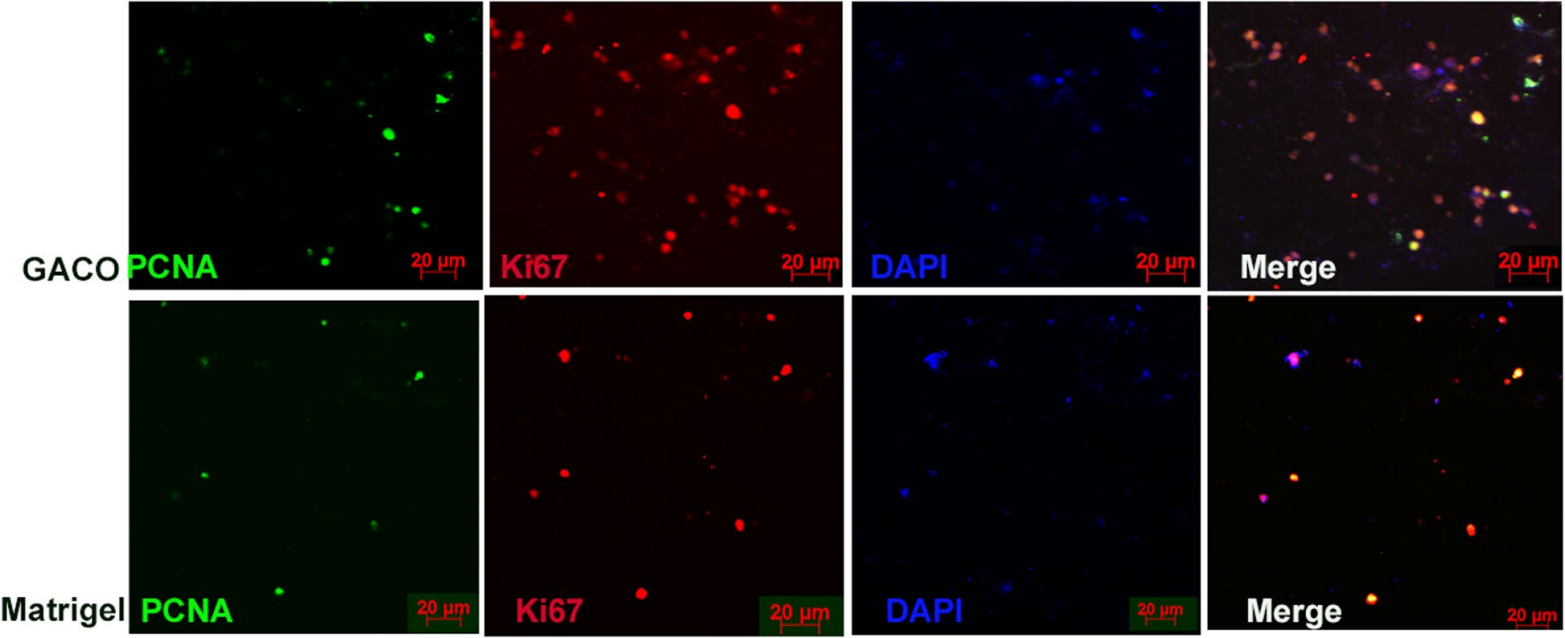
Panel showing staining of organoid sections from the GACO gel and matrigel. Cells appear to be positive for Ki67 and PCNA among the clusters of the cells

The cells at the pancreatic progenitor stage were assembled and trapped into matrigel and incubated in the organoid medium for 10–30 days. The pancreatic progenitor cells were able to form cyst like structures and morphology of the organoids was irregular. The organoids could be re-expanded after trypsinization from very few cells (Fig. 4E) or by mechanical dissociation (Fig. 4F, G). Organoids were seen expressing markers of endocrine and exocrine cells (Fig. 5, 6 and 7) as seen by immunohistochemistry.

GACO gels were designed by crosslinking the collagen with the oxidized gum arabic, these gels are stable and biocompatible to a range of cells (Sarika et al. 2014; Rohit et al. 2021). These gels can be fabricated by changing oxidation level of gum arabic and collagen concentration to suit the need of the cells (Rohit et al. 2021, Uday Chandrika et al. 2021). Organoids were assembled in various concentrations of collagen (0.25% to 1%) that were crosslinked with gum arabic oxidized to 5%. Depending on the cell viability we decided to continue with GACO gel with 0.25% collagen (0.25% C) [Table 3]. Cells in the higher collagen concentration did not appear to aggregate and remained isolated within the scaffold.

**Table 3:** Viability of cells in GACO scaffolds

Collagen concentration	GACO Oxidation level	MTT value mean	SD
0.25	5%	0.2125	0.0074
0.5	5%	0.0901	0.0536
1	5%	0.1256	0.0667
Control	–	0.3627	0.0681

Islet organoids were trapped in the matrigel, collagen and GACO gel (0.25% C) and compared for the marker appearance and the functional tests. The most stable among these were the GACO organoids. The cell cluster grew the best in the matrigel and the GACO hydrogels (with 0.25% C). Organization of the cells was clustered for islet cells having mostly beta cells that are positive for insulin, amylin and NGN3 with rare positive cells for glucagon and somatostatin (Fig. 6). At the periphery of these clusters of islet cells, one could also locate few cells that are acinar like positive cells for carboxypeptidase and pancreatic amylase (Fig. 6). GACO and the matrigel showed presence of cells with proliferation capacity as the cells stained positive for both PCNA and Ki67 (Fig. 7). Collagen scaffolds were most unstable for any kind of analysis, even the stability of the organoid was compromised while handling.

**Fig. 8:**
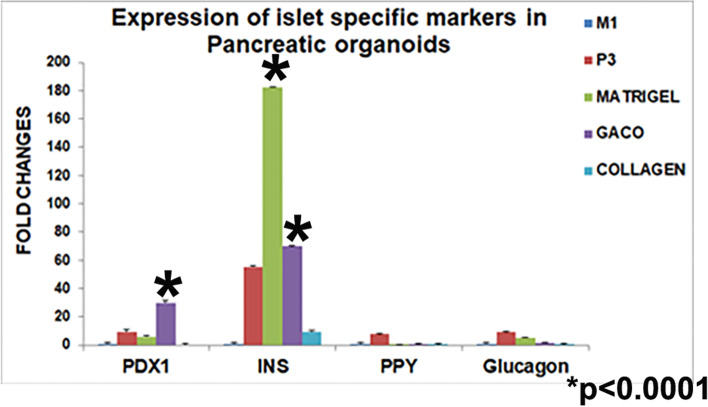
qPCR analysis of islet specific genes in collagen, GACO and matrigel in comparison to M1. *PDX1*, *Insulin*, *PPY* and *glucagon* expression was seen in GACO and matrigel. Collagen entrapped cells did not show detectable levels of any gene expression. *Insulin* was higher in GACO gels. Expression of markers in the organoids is significantly higher as compared to stage P3 cells

Gene expression analysis of various stages up to the pancreatic progenitor stage and after the organoid formation also revealed stage specific changes in the gene expression. The organoids assembled in matrigel, GACO gel and collagen were compared for the quantitative expression of islet specific genes. *Insulin* and *PDX1* showed significantly higher levels of expression as compared to the parent cell line M1 and the pancreatic progenitor stage 3 cells [*p* < 0.001] (Fig. 8). *PPY* and *glucagon* expression also could be seen in the organoids. Expression of the islet marker genes in organoids was significantly higher as compared to the pancreatic progenitor cells (PP) [*p* < 0.05]. The organoids encapsulated in the collagen did not have a detectable fold change in expression of most of the markers. The samples for collagen entrapped organoids were collected by centrifuging the contents of the dish that was otherwise difficult to handle.

### Glucose induced insulin production

**Fig. 9:**
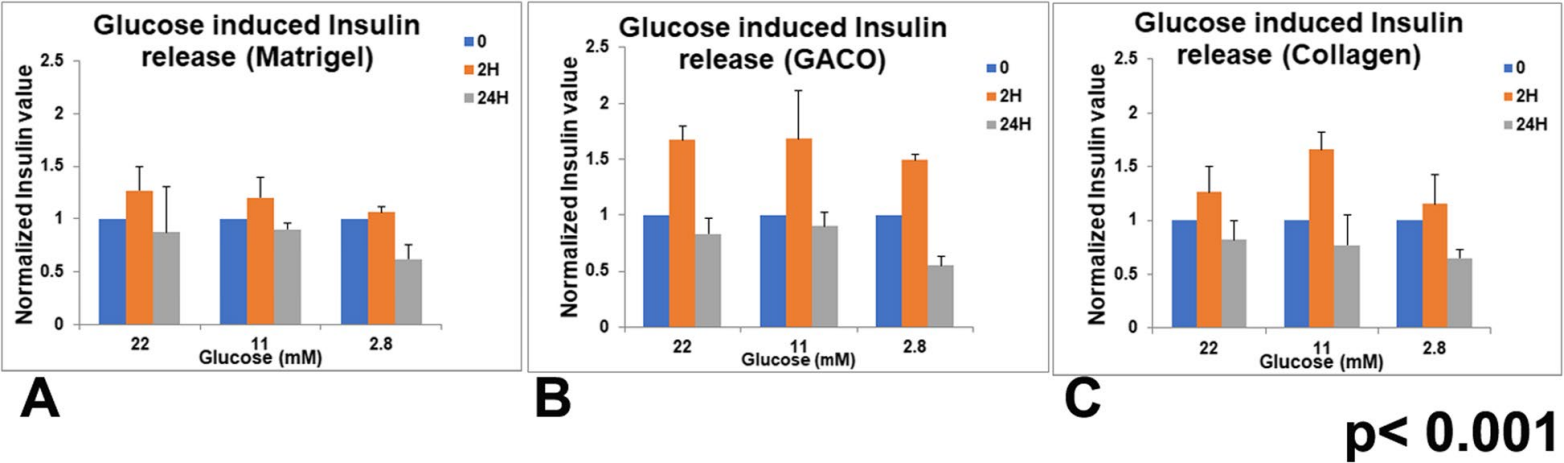
Glucose stimulated Insulin release in the organoids. Supernatant was collected at 2 h and 24 h after addition of glucose to the medium. The organoids embedded in matrigel (**A**), GACO gel (**B**) and collagen (**C**) also show increased insulin release at all concentrations of added glucose though the response is not seen in 24 h. GSIS was highest at 22 mM of glucose

The most important function of islets is the secretion of insulin in response to glucose exposure. The organoids were checked for insulin release around after 20 days. Supernatant collected from the organoids incubated individually in non-adherent round bottom wells were analyzed for insulin content and expressed per mg protein. After overnight starvation in glucose free medium, Organoids were incubated in different glucose levels and supernatant was collected after 2 and 24 h. After 2 h of glucose exposure, 25% release of insulin was observed upon addition of 22 mM glucose though the effect seen was not as pronounced at lower glucose levels (Fig. 9).

The organoids enclosed in different scaffolds were also compared for the insulin release. In all the three matrices, organoids remained responsive to glucose stimulation, at all glucose concentrations (Fig. 9A-C). Insulin release could be observed after 2 h of glucose stimulation in all the organoids in different matrices. Release was comparatively higher in GACO (75%) and collagen gels (20%) at 22 mM glucose exposure. At lower concentrations of glucose also one could see some insulin release. Intra-assay statistics did not show significant differences but comparison between different gels did show a significant difference in insulin release assay. The insulin levels did not remain high at 24 h of glucose exposure.

## Discussion

The protocol adopted for the generation of PP cells and finally the organoids resulted in efficient generation of the cells at each defined stage. The results reveal that the GACO scaffold used as matrix for assembled organoid was able to support the organoids for above 30 days and retained the identity of cells and remained functional. The GACO scaffolds were quite stable as well.

The factors used for differentiation of the iPSC cells induced efficient differentiation of pancreatic progenitor cells that could subsequently form insulin-producing cells in islet like structures. Each stage could be ascertained by immunohistochemistry and qPCR studies. Starting with iPSC, we could stage the endoderm formation that showed enhanced expression of SOX17, PAX6 and GATA4; these cells under the influence of EGF could give rise to foregut lineage that expressed PDX1. This marks the emerging pancreatic lineage (McCracken et al. 2014) and is also essential for it. Under the serial influence of other molecules like noggin, ascorbic acid and PKC inhibitors we see the pancreatic progenitor cells emerging which show NKX6.1, along with PDX1 by the end of the treatment cells are expressing all PP markers (Nostro et al. 2015).

The pancreas progenitor cells generated could be assembled into organoids successfully. The pancreatic progenitor and the beta cells are known to display self-clustering dynamics even in ex vivo culture (Puri and Hebrok 2007). The organoids developed had beta cells marker expression along with few rare cells expressing glucagon and somatostatin. The organoids were also able to show glucose stimulated insulin release. Once the process of organoid formation was established the organoids were compared in different matrices.

GACO scaffolds were found to be quite effective for seeding the assembled pancreatic progenitor cells. Cells were found to be comparable in the expression of islet specific markers as in the matrigel assembled cells. Quantitative analysis of the gene expression also showed comparable expression of the markers. The GACO scaffold organoids were responsive to glucose stimulated insulin release; in fact the response was higher in terms of insulin released per mg of protein. It could actually be much higher as the scaffold is very rich in protein as well. It proved to be suitable also in terms of diffusibility of the nutrients and metabolites as we could measure the insulin levels in 2 h in the supernatant in response to glucose. Release of insulin into the supernatant supports the notion that the cells are able to access the media and surroundings while maintaining a stable aggregate structure. The GACO preparations in earlier studies have been shown to be stable (Sarika et al. 2014; Rohit et al. 2021; Uday Chandrika et al. 2021) and biocompatible. Their stability is reflected here also in terms of culture handling, where collagen embedded organoids were not able to hold the cluster of cells and appeared dispersed; the GACO scaffolds remained intact with structure and function. The pancreatic progenitor cells seeded into the GACO scaffolds were stable and viable for 30 days. Just plain collagen gels was found to be lacking in the holding strength, the scaffolds would collapse and the cells dispersed as reported earlier (Wang and Ye 2009). Others have used collagen in combination with the matrigel (Wang et al. 2017). We report an efficient generation of the islet organoids using GACO scaffold that eliminates the use of serum as well as matrigel.

The organoids developed from pluripotent stem cells are powerful means to generate 3D models of human organs and of their dysfunctions in case derived from diseased iPSC or with genetically altered cell lines. Lack of certain cell types such as mesenchymal/stromal cells, blood vessels and nerves that are required for the complete organ function can be included during the assembly to develop a long sustaining organoid. GACO scaffolds were used after the pancreatic progenitor stage in this study but could be used for starting differentiation of iPSC.

HiPSC can be differentiated into glucose responsive islet like structures using GACO gels. The data obtained might be useful for using this model system for understanding mechanism of insulin secretion in the human pancreatic cells or for drug screening. Further studies with the animal models in vivo would help us elucidate the significance of these gels in relation to restoration and sustenance of endocrine pancreatic function.

## Conclusions

Collagen crosslinked with oxidized gum arabic can be used for formation of pancreatic organoids from iPSCs, the islet clusters obtained showed presence of differentiated cells and were able to release insulin upon glucose stimulation.

## Data Availability

The data is available with Dr. Shashi Singh (shashis@csirccmb.org).
